# Reversal of Polymicrobial Biofilm Tolerance to Ciprofloxacin by Blue Light plus Carvacrol

**DOI:** 10.3390/microorganisms9102074

**Published:** 2021-10-01

**Authors:** Yongli Li, Mei X. Wu

**Affiliations:** Wellman Center for Photomedicine, Massachusetts General Hospital, Department of Dermatology, Harvard Medical School, 50 Blossom Street, Boston, MA 02114, USA; YLI109@mgh.harvard.edu

**Keywords:** polymicrobial biofilm, blue light (BL), carvacrol (Ca), biofilm tolerance, ciprofloxacin (CIP), membrane permeability

## Abstract

Chronic wound infections are often caused by multi-species biofilms and these biofilm-embedded bacteria exhibit remarkable tolerance to existing antibiotics, which presents huge challenges to control such infections in the wounds. In this investigation, we established a polymicrobial biofilm composed of *P. aeruginosa*, *S. aureus*, *K. pneumoniae*, and *A. baumannii*. We tested a cocktail therapy comprising 405-nm blue light (BL), carvacrol (Ca), and antibiotics on the multispecies biofilm. Despite the fact that all strains used to form the biofilm were susceptible to ciprofloxacin (CIP) in planktonic cultures, the biofilm was found to withstand ciprofloxacin as well as BL-Ca dual treatment, mainly because *K. pneumoniae* outgrew and became dominant in the biofilm after each treatment. Strikingly, when ciprofloxacin was combined with BL-Ca, the multispecies biofilms succumbed substantially and were eradicated at an efficacy of 99.9%. Mechanistically, BL-Ca treatment increased membrane permeability and potentiated the anti-biofilm activity of ciprofloxacin, probably by facilitating ciprofloxacin’s entrance of the bacteria, which is particularly significant for *K. pneumoniae*, a species that is refractory to either ciprofloxacin or BL-Ca dual treatment. The results suggest that bacterial membrane damage can be one of the pivotal strategies to subvert biofilm tolerance and combat the recalcitrant multispecies biofilms.

## 1. Introduction

Biofilm is a common bacterial lifestyle in nature. These bacterial aggregates have been found in a variety of niches ranging from pond water to catheters, from chronic wounds to airway mucus. Biofilm-embedded bacteria are well-protected from antibiotic killing as compared with their planktonic counterparts. Phenotypic tolerance to antibiotics is reversible sometimes following biofilm dispersion. Several hypotheses have been proposed to address increased antibiotic tolerance of biofilms over their planktonic counterparts [1,2,3]: i.e., (i) matrix barrier retards penetrations of cationic compounds like aminoglycosides [4]; (ii) environmental stresses such as hypoxia, nutrient deprivation, or accumulation of wastes reduce bacterial growth rates and induce antibiotic indifference; (iii) persister cells have their essential machinery such as translation shutting down to outlast antibiotic hitting [5]; (iv) robust antioxidant systems can detoxify antibiotic-induced Reactive Oxygen Species (ROS) [6]; and (v) efflux pumps are up regulated [7].

Biofilm accounts for over 60% of human infections [8], many of which are polymicrobial [9,10,11]. Chronic wounds such as diabetic foot ulcers are filled with polymicrobial biofilm, where *Staphylococcus*, *Pseudomonas*, *Escherichia*, *Klebsiella*, and *Acinetobacter* have been frequently isolated together [12]. Multi-species interactions may modulate community tolerance to antibiotics, making it extremely difficult to treat. For instance, after gentamicin treatment, *P. aeruginosa* in a wound-like polymicrobial biofilm showed a two-fold higher survival than in the mono-species biofilm [13]. In cystic fibrosis sputum, *P. aeruginosa* can enhance *S. aureus* tolerance to aminoglycoside antibiotics by inducing small-colony variants (SCVs) [14]. To grow biofilms in evaluation of various antimicrobial modalities, several artificial biofilm models have been developed [15] including the microtiter plate method, CDC biofilm reactors [16], drip-flow reactors [17], etc. The Lubbock Chronic Wound Pathogenic Biofilm (LCWPB) medium is a sophisticated model that aims to mimic wound conditions where plasma, erythrocytes, and chopped-meat-based medium are presented [13,18,19]. If a coagulase-positive *S. aureus* is provided in LCWPB, the *S. aureus* can promote plasma coagulation and provide a tridimensional scaffold that accommodates multi-species bacteria, a detail study of which can be found [20].

A few novel modalities are being explored individually or as adjuncts to conventional antibiotics to combat the recalcitrant biofilm [7,21,22,23]. Our laboratory previously demonstrated the antimicrobial/anti-biofilm potential of blue light (BL) and carvacrol (Ca) dual therapy against *P. aeruginosa*, *S. aureus*, and *A. baumannii* [24]. Blue light at 405 nm excited endogenous porphyrins and induced ROS eruption of the bacteria [25,26]. Carvacrol is a phenolic monoterpenoid commonly presented in medicinal essential oils from aromatic plants. The antimicrobial property of Ca has been widely appreciated, while a “pro-photosensitizing” nature of Ca was discovered just recently [24,27]. Briefly, Ca is oxidized to thymoquinone by the BL mediated ROS; thymoquinone happens to be a photosensitizer which absorbs 405-nm BL and dramatically augment the oxidative burst. The overwhelming ROS indiscriminately damages bacterial components and eventually kills the cells. BL and Ca synergistically kill planktonic and biofilm-embedded cells of *P. aeruginosa*, *S. aureus*, and *A. baumannii*, respectively. However, only mono-species biofilms were tested in those experiments. Moreover, other wound-associated bacteria such as *E. coli* and *K. pneumoniae* do not succumb to the BL-Ca dual therapy in part because they produce a low or moderate level of metal-free tetrapyrrole macrocycles [28].

We hypothesize that the BL-Ca dual therapy can be combined with antibiotics to more sufficiently eradicate polymicrobial biofilms. To this end, a polymicrobial biofilm model comprising four common wound pathogens (*S. aureus*, *K. pneumoniae*, *A. baumannii*, *P. aeruginosa*) was established, which was sufficiently exterminated by a combination of BL-Ca and ciprofloxacin but not by either alone. The results demonstrate significant reversal of the biofilm-associated tolerance to antibiotic ciprofloxacin probably owing to a bacterial membrane permeabilizing action of BL-Ca treatment.

## 2. Materials and Methods

### 2.1. Light Source, Compounds, and Microorganisms

A light-emitting diode (Thorlabs, Newton, NJ, USA) with peak emission at 405 nm and a full width at half maximum of 12.5 nm was used. The irradiation was adjusted to 55 mW/cm^2^ by a PM100D power/energy meter (Thorlabs). Carvacrol (282197-10G) was purchased from Sigma-Aldrich and prepared at 100 mg/mL in dimethyl sulfoxide, ciprofloxacin hydrochloride monohydrate (Alfa Aesar, J61970) at 32 mg/mL in sterile water, and tetracycline (Alfa Aesar, J61714) at 64 mg/mL in dimethyl sulfoxide. *Staphylococcus aureus* IQ0064, *Escherichia coli* IQ0245, and *Klebsiella pneumoniae* IQ0035 were multidrug-resistant isolates obtained from the San Antonio Military Medical Center, USA [29]. *Acinetobacter baumannii* ATCC 17978 and *Pseudomonas aeruginosa* strain HER-1018 [PAO1] (ATCC BAA-47) were also used in this study. The bacteria were cultured in Brain Heart Infusion (BHI) medium (BD BBL^TM^; B11059) with shaking at 250 rpm, unless otherwise indicated.

### 2.2. Anti-Planktonic Bacteria Assay

Overnight cultures of *P. aeruginosa* or *K. pneumoniae* planktonic bacteria were diluted with PBS to obtain OD_600_ readings of 0.01 or 0.05, respectively. Aliquots of 100 µL bacterial suspension per well with an indicated carvacrol dose were transferred to a transparent 96-well plate (Corning, Tewksbury, MA, USA) and exposed to BL until a designated irradiance was reached (3 min irradiation equals 10 J/cm^2^). The numbers of survived bacteria were determined by a microdilution plating method and CFU counts with a detection limit of 100 CFU/mL.

### 2.3. Polymicrobial Biofilm

Multi-species biofilm was grown in the Lubbock chronic wound pathogenic biofilm (LCWPB) medium [18]. The LCWPB medium was formulated with 48% Bolton Broth (NEOGEN, NCM0094A), 50% Bovine Plasma (HemoStat, SBP500), and 2% Laked Horse Blood (HemoStat, LHB100). To make the five-species biofilm, overnight *S. aureus*, *E. coli*, *K. pneumoniae*, and *A. baumannii* cultures were individually diluted 1000 times in BHI, which had approximately 10^7^ CFU/mL each. Overnight culture of *P. aeruginosa* was typically diluted 5000 times to obtain 10^7^ CFU/mL. Aliquots of each diluted bacteria at 10 µL were inoculated into 6 mL LCWPB medium; a 1 mL pipette tip (Fisher Scientific, Waltham, MA, USA) was ejected into the medium to provide a surface for biofilm establishment. The tubes were then incubated at 37 °C with slow shaking at 100 rpm for 48 h. To make the four-species biofilm, a mixture of *S. aureus*, *K. pneumoniae*, *A. baumannii*, and *P. aeruginosa* at a starting ratio of 1:1:1:1 was used to inoculate the LCWPB medium.

### 2.4. Quantitative PCR (qPCR) to Measure Biofilm Bacterial Composition

Species-specific primer pairs were designed to target a unique gene in the respective bacterial genome (Table 1). Genomic DNA (gDNA) of *S. aureus*, *E. coli*, *K. pneumoniae*, *A. baumannii*, and *P. aeruginosa* was individually extracted from planktonic cells by the PureLink Genomic Mini Kit (Invitrogen, Waltham, MA, USA). qPCR was carried out with 10 ng gDNA using a Power SYBR™ Green PCR Master Mix (Applied Biosystems™, Waltham, MA, USA) in the following reaction cycle: 95 °C for 10 min, and 40 cycles of 95 °C for 10 s, 60 °C for 10 s, and 72 °C for 15 s.

The bacterial composition of a biofilm was determined using a modified method as described by Sun et al. [18]. Briefly, the entire biofilm was stripped off from the pipette tip, washed twice with PBS, and then disrupted by Lysing Matrix D (MP Biomedicals, Solon, OH, USA). gDNA of the resuspended biofilms-associated cells was extracted, diluted, and added to a qPCR reaction (10 ng/reaction). Ct values of the target genes were obtained after qPCR reactions. The Ct of the 16S rRNA gene was subtracted from the Ct of the target gene to produce the ΔCt. The ΔCt value of *P. aeruginosa* was subtracted from every other sample to produce the ΔΔCt value. 2^−ΔΔCt^ was taken for every sample and used to calculate the relative ratios of each species.

### 2.5. Anti-Biofilm Assays

A 2-day-old polymicrobial biofilm (four-species biofilm) was cut into 3×6-mm pieces each about 10 mg containing around 10^7^ CFU. In some experiments, the biofilms were ground for 10 min using Lysing Matrix D and a FastPrep disruption device (MP Bio) prior to treatment. For BL-Ca dual treatment (BL^+^Ca^+^), the biofilm was placed in a 24-well plate and submerged in 2 mL PBS with 125 µg/mL Ca and then exposed to BL for an indicated length. As a control (BL^−^Ca^−^), the biofilm was soaked in PBS and kept from light. For antibiotic treatment, the biofilm was submerged in BHI supplemented with 10 µg/mL ciprofloxacin or 40 µg/mL tetracycline and incubated at 37 °C for 5 h. As a control (non-antibiotic treatment), the biofilm was kept in cold PBS. After treatments, the biofilms were washed three times with fresh PBS and disrupted by Lysing Matrix D. The number of survived bacteria was determined by a microdilution plating method and CFU counts with a detection limit of 100 CFU/mL. After CFU enumeration, colonies/lawns of the lowest dilution were scraped, resuspended in PBS and then subject to qPCR to determine the composition of the survived bacteria. Synergy between BL-Ca and antibiotics was assessed by S-values based on the Bliss Independence model according to the following formula [30]: S-value = (Log^CFU^_BL+Ca_/Log^CFU^_Ctrl_) × (Log^CFU^_antibiotic_/Log^CFU^_Ctrl_) − (Log^CFU^_BL+Ca+antibiotic_/Log^CFU^_Ctrl_). Log^CFU^_BL+Ca_, Log^CFU^_antibiotic_, Log^CFU^_BL+Ca+antibiotic_, or Log^CFU^_Ctrl_ is the number of survived bacteria after a treatment of BL-Ca dual treatment, antibiotic mono-treatment, the cocktail, or negative control, respectively.

### 2.6. Measure Bacterial Membrane Permeability

Overnight culture of *P. aeruginosa* or *K. pneumoniae* was diluted to obtain OD_600_ reading of 0.1 or 0.5, respectively. Aliquots of 1 mL bacterial suspension were treated with PBS (BL^−^Ca^−^), 125 µg/mL carvacrol (BL^−^Ca^+^), 80 J/cm^2^ blue light (BL^+^Ca^−^), or both (BL^+^Ca^+^). The bacteria were also treated with 70% isopropyl alcohol for 24 min as positive controls. The treated samples were stained by 50-µM Propidium Iodide for 15 min, washed, and fixed by 1% paraformaldehyde for 15 min prior to analysis of PI fluorescence (ex/em:535/617 nm) on a microplate spectrophotometer (Molecular Devices) or flow cytometry (BD Biosciences). All flow cytometric data were analyzed by FlowJo software.

### 2.7. Statistical Analysis

All statistical analyses were performed using GraphPad Prism 9.0 (GraphPad Prism, San Diego, CA, USA). Statistics significance was analyzed with two-tailed Student’s t test between two groups or one-way ANOVA for multiple group comparisons. *p* values of <0.05 were considered significant. Data were presented as mean ± standard deviation (SD).

## 3. Results

### 3.1. P. aeruginosa Dominate the Five-Species Polymicrobial Biofilm

**Figure 1:**
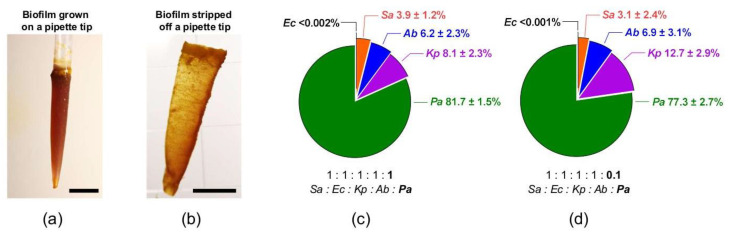
Bacterial composition of polymicrobial biofilms. (**a**) A typical 2-day-old polymicrobial biofilm grown in the LCWPB medium. The five-species biofilm was attached to the surface of a 1 mL pipette tip. (**b**) The opaque and thin biofilm stripped from the pipette tip. Scale bar, 1 cm for (**a**,**b**). (**c**) Species composition of the polymicrobial biofilm (*n* = 6). (**d**) Species composition of the polymicrobial biofilm (*n* = 3). The ratios below indicate the initial share of each bacterial inoculum. *Sa*: *S. aureus*; *Ec*: *E. coli*; *Kp*: *K. pneumoniae*; *Ab*: *A. baumannii*; *Pa*: *P. aeruginosa*.

Five pathogens that are commonly found in chronic wounds including Gram-negative *E. coli*, *K. pneumoniae*, *A. baumannii*, *P. aeruginosa*, and Gram-positive *S. aureus* were mixed and incubated for 48 h in Lubbock Chronic Wound Pathogenic Biofilm (LCWPB) medium. Bacteria could attach to the provided pipette tip and initiated biofilm establishment. Figure 1a showed a typical polymicrobial biofilm formed on a 1 mL pipette tip, which appeared as an opaque veil-like structure after peeled off (Figure 1b). The thickness was less than 1 mm and the yellowish colonies across the biofilm was most ascribed to *S. aureus*, while colonies of other species were invisible.

Species-specific primers were employed to decipher the bacterial compositions of the biofilms. Each primer set was designed to target a species-specific gene as detailed in Table 1. The specificity and cross-reactivity of each primer pairs was confirmed by endpoint PCR and qPCR. For all primer pairs, Ct values plotted against genomic dilutions (10 to 0.0077 ng) displayed a high linearity (R^2^ > 0.99). The amplification efficiencies of primers were comparable and could be used to unbiasedly determine the individual share of the five species within the biofilm community.

The initial ratio of bacterial inoculum was set to 1:1:1:1:1 in attempt to grow biofilms with equal shares for all five individual species. This was apparently impossible as the growth of each species varied drastically in the biofilm. *P. aeruginosa* was the dominating species accounting for 81.7%, followed by *K. pneumoniae* (8.1%), *A. baumannii* (6.2%), *S. aureus* (3.9%), and *E. coli* (<0.002%) (Figure 1c). *E. coli* IQ0245 struggled to find its niche in this multi-species community. We tried different ratios and failed to establish a more balanced biofilm. The best effort ended to lower the initial *P. aeruginosa* by 10 times while keeping the other inocula unaltered, which still did not dramatically affect the overall balance of the biofilm community (Figure 1d). Having dropped only by 4.4%, *P. aeruginosa* remained predominant (77.3%) and the *P. aeruginosa*-conceded shares were picked up by *K. pneumoniae*, which grew from 8.1% to 12.7%. At the same time, *A. baumannii* (6.9%), *S. aureus* (3.1%), and *E. coli* (<0.001%) stayed put.

### 3.2. Polymicrobial Biofilms Tolerate Ciprofloxacin

As shown in Figure 1c,d, *E. coli* IQ0245 appeared to be negligible in the five-species biofilm community and thus removed from subsequent studies. In fact, leaving out the *E. coli* inoculum resulted in a four-species biofilm with a similar bacterial composition as the five-species biofilm. Table 2 detailed the Minimal Inhibitory Concentrations (MICs) of ciprofloxacin and tetracycline against the planktonic pathogens that were used to form the four-species biofilm. All strains were sensitive to both antibiotics. This four-species polymicrobial biofilm was an excellent model to probe the difference of antibiotic susceptibility between the free-living and the biofilm-associated bacteria.

**Figure 2:**
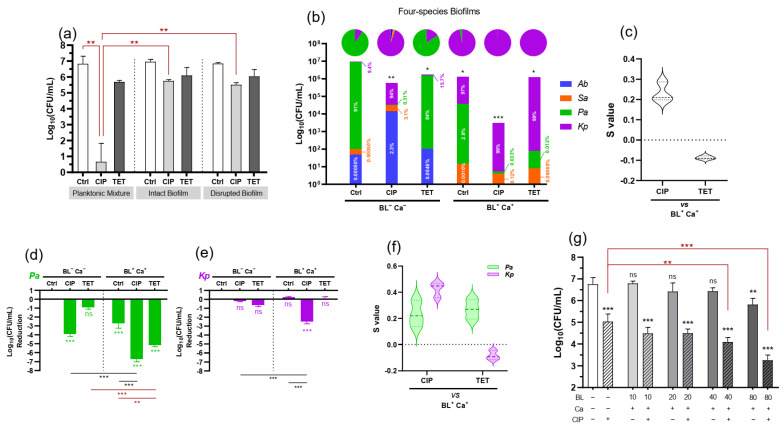
BL-Ca treatment enhances ciprofloxacin potency on polymicrobial biofilms. (**a**) Biofilm-associated tolerance to ciprofloxacin (CIP) or tetracycline (TET) as compared with the planktonic mixture with a similar composition. The bacteria either in intact or disrupted biofilm or planktonic culture were exposed to CIP (10 µg/mL) or TET (40 µg/mL) for 5-h in BHI medium, after which bacteria (Log_10_CFU/mL) survived the treatment were determined. (**b**) Stacked bar chart showing survived bacteria after different treatments. Pie charts above each column depicted corresponding species compositions in linear scale. (**c**) Synergy of BL-Ca treatment and antibiotics on biofilm survival. (**d**,**e**) Log reductions of biofilm-associated *P. aeruginosa* (**d**) and *K. pneumoniae* (**e**) after different treatments. (**f**) Synergy of BL-Ca treatment and antibiotics on the survival of embedded *P. aeruginosa* or *K. pneumoniae* determined by S values as detailed in Materials and Methods. 0 ˂ S ˂ 1 indicates a synergistic interaction, whereas S ˂ 0 indicates an antagonistic interaction. (**g**) Increasing BL irradiance could enhance the potentiation of CIP on polymicrobial biofilms. *Sa*: *S. aureus*; *Ec*: *E. coli*; *Kp*: *K. pneumoniae*; *Ab*: *A. baumannii*; *Pa*: *P. aeruginosa*. *** *p* < 0.001; ** *p* < 0.01; * *p* < 0.05; and ns, no significance.

The antimicrobial activities of ciprofloxacin (10 µg/mL) and tetracycline (40 µg/mL) were tested against the planktonic mixture of *P. aeruginosa* (80%), *K. pneumoniae* (10%), *A. baumannii* (6%), and *S. aureus* (4%) in mimicking the composition of the polymicrobial biofilms described in Figure 1. The bactericidal ciprofloxacin rapidly wiped out the planktonic bacteria in 5 h while reducing merely 1.20 Log biofilm-encased bacteria (Figure 2a). Tolerance of the biofilm to ciprofloxacin was little improved even after mechanical disruption of the biofilms. Ciprofloxacin killed only 1.33 Log bacteria released from the disrupted biofilm. The observations were consistent with a significantly higher tolerance of biofilm-encased bacteria to ciprofloxacin and such tolerance did not result from the matrix barrier. In contrast, the bacteriostatic tetracycline was not effective irrespective of whether the bacteria were in planktonic culture or in intact or disrupted biofilms.

### 3.3. BL-Ca Dual Treatment Potentiates Anti-Biofilm Activities of Ciprofloxacin and Reshapes the Biofilm Community

Viable counts in intact polymicrobial biofilm (10 mg) were reduced by 1.20 or 0.84 Log only after ciprofloxacin (10 µg/mL) or tetracycline (40 µg/mL) challenges, respectively (Figure 2b). Intriguingly, if the biofilm was pre-treated with 80 J/cm2 BL and 125 µg/mL Ca, another 2.32 Log reduction was observed after ciprofloxacin challenge. The total anti-biofilm efficacy of the BL-Ca-CIP cocktail was 3.52 Log reduction, which was greater than 99.9% eradication of biofilm-associated cells. The potentiating effect of BL-Ca dual treatment on ciprofloxacin was synergistic, confirmed by the positive S value (0.23) (Figure 2c). BL irradiance could dose-dependently potentiate ciprofloxacin’s efficacy (Figure 2g). At 40 and 80 J/cm2, BL in combination with Ca further enhanced the killing of ciprofloxacin by 1.0 and 1.8 Log, respectively. However, pre-treatment of BL-Ca therapy did not render additional susceptibility to the tetracycline challenge (Figure 2b). Neither was there any synergistic interaction between the dual therapy and tetracycline (Figure 2c).

The untreated biofilm was *P. aeruginosa*-dominated (91%) with *K. pneumoniae* (9.4%) as the second most resident (Figure 2b). Tetracycline challenge at 40 µg/mL did not affect much of the composition; the biofilm was still dominated by *P. aeruginosa* (84%) and then *K. pneumoniae* (16%). Of note, the ciprofloxacin challenge at 10 µg/mL greatly diminished *P. aeruginosa* from 84% to 0.31%, concurrent with rising proportions of *K. pneumoniae* to 94% from 16%, *S. aureus* to 3.1% from 0.0005%, and *A. baumannii* to 2.3% from 0.0005% when compared in the presence vs. absence of ciprofloxacin challenge (Figure 2b). The results strongly argue that in the multi-species biofilm community, killing only CIP-susceptible species like *P. aeruginosa* would facilitate other species to outgrow making the treatment futile and more difficult to manage subsequently. BL-Ca dual treatment specifically targeted *P. aeruginosa*, *S. aureus*, and *A. baumannii* cells and the treatment transformed the biofilm into a *K. pneumoniae*-dominated (97%) community containing some vestigial *P. aeruginosa* (2.9%). On top of the BL-Ca dual treatment, either ciprofloxacin or tetracycline could further remove the lingering *P. aeruginosa* cells and the treated biofilm became virtually single-species (*Kp* > 99.8%).

### 3.4. Biofilm-Associated P. aeruginosa or K. pneumoniae Respond Differently to BL-Ca Treatment

Either *P. aeruginosa* or *K. pneumoniae* was the predominant species after whichever treatment was given to the biofilm (Figure 2b). Accordingly, individual Log reduction of *P. aeruginosa* or *K. pneumoniae* was calculated to compare the killing effect of different modalities at the species level (Figure 2d,e). *P. aeruginosa* was susceptible to all modalities except tetracycline challenge (Figure 2d). In marked contrast, the biofilm-encased *K. pneumoniae* could tolerate all modalities except the BL-Ca-CIP cocktail (Figure 2e). Specifically, BL-Ca-CIP significantly reduced (2.45 Log) the viable *K. pneumoniae*, while none of the CIP, TET, BL-Ca, or BL-Ca-TET alone was effective. It was clear that BL-Ca dual treatment potentiated both ciprofloxacin and tetracycline on *P. aeruginosa* as suggested by the positive S values (Figure 2f; green). However, BL-Ca worked synergistically with the bactericidal ciprofloxacin rather than the bacteriostatic tetracycline to kill the biofilm-embedded *K. pneumoniae* (Figure 2f; magenta).

### 3.5. BL-Ca Treatment Permeabilizes Bacterial Membranes

To pinpoint the mechanism underlying the potentiation of BL-Ca dual treatments on CIP bactericidal activity against the multi-species biofilm, flow cytometric analysis of propidium iodide (PI)-stained planktonic bacteria was employed to analyze bacterial membrane integrity, on the assumption that ROS generation following BL-Ca dual treatment could damage bacterial envelops facilitating ciprofloxacin to enter the bacteria on the basis of our previous studies [24,27]. The membrane-attacking property of carvacrol was also reported, albeit to a much lesser extent [29]. In comparison with BL^−^Ca^−^ controls, the relative PI fluorescence of *P. aeruginosa* increased by 1.1- and 2.8-fold after treatment with BL^−^Ca^+^ or BL^+^Ca^−^ regimen, respectively. Notably, PI intensity of *P. aeruginosa* took off and increased by 15-fold after BL^+^Ca^+^ treatment, approaching almost complete membrane disruption at levels only slightly lower than the PI intensity induced by isopropyl alcohol. BL and Ca likely acted synergistically in disrupting the *P. aeruginosa* outer/cytoplasmic membranes.

**Figure 3:**
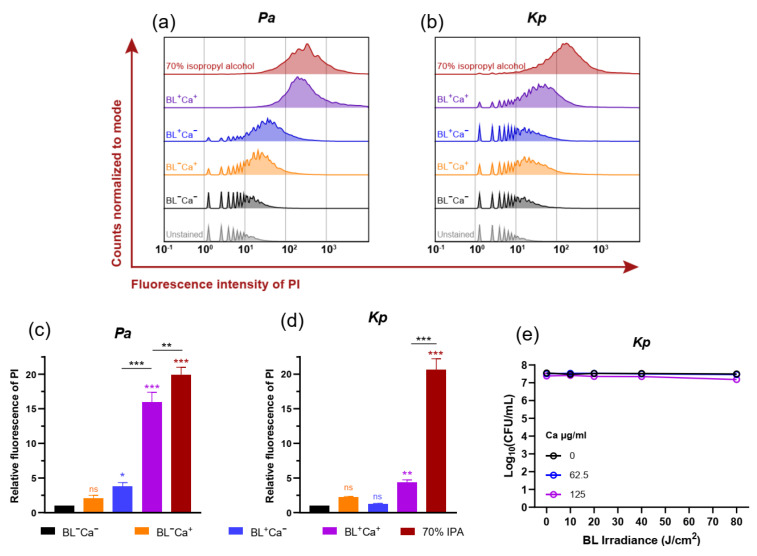
BL-Ca treatment permeabilizes bacterial membranes. (**a**,**b**) Representative histograms showing PI fluorescence intensities of *P. aeruginosa* (a) and *K. pneumoniae* (b) after different treatments. (**c**,**d**) Mean PI fluorescences of *P. aeruginosa* (**c**) and *K. pneumoniae* (**d**) after different treatments relative to the negative control (BL-Ca-). BL, 80 J/cm^2^ and Ca, 125 µg/mL. (**e**) Survivals of *K. pneumoniae* IQ0035 after different treatments. *Kp*: *K. pneumoniae*; *Pa*: *P. aeruginosa*. Results are presented as Mean ± SD of three independent experiments. *** *p* < 0.001; ** *p* < 0.01; * *p* < 0.05; and ns, no significance.

It was apparent that BL alone did not cause as much damage to *K. pneumoniae* membranes as it did to *P. aeruginosa*. After BL^+^Ca^−^ treatment, the “discrete peaks” at 10^0^ to 10^1^ were still prominent in the *K. pneumoniae* sample, indicating a significant proportion of unstained bacteria due to the healthy membranes (Figure 3b,d; blue trace and bar). BL^+^Ca^+^ treatment could compromise the *K. pneumoniae* outer/cytoplasmic membranes, as suggested by the shrunk “discrete peaks” and the significantly increased PI fluorescence (3.4 folds) (Figure 3b,d; magenta trace and bar). However, *K. pneumoniae* permeability induced by BL^+^Ca^+^ was much lower than that caused by isopropyl alcohol, probably resulting from impaired membrane integrity rather than complete disruption. In line with this, BL^+^Ca^+^ combinations did not seem to affect *K. pneumoniae* viability at Ca concentrations ≤ 125 µg/mL and BL fluence ≤ 80 J/cm^2^ (Figure 3e). Under the treatment conditions, *K. pneumoniae* could withstand the membrane injury and survive BL^+^Ca^+^ challenges. Yet, the membrane injury may enable ciprofloxacin to enter the cells for bactericidal action of the antibiotics.

## 4. Discussion

Our data support that BL-Ca dual therapy could be further combined with conventional antibiotics to reverse biofilm-associated tolerance. Biofilm infections can linger for months or years because of their remarkable tolerance to antimicrobials, disinfectants, and host immune responses. Intensive and aggressive antibiotic treatments are often required to control biofilm dissemination, which however can be toxic or far beyond the limitation of renal and hepatic functions [32]. The proposed cocktail therapy consisting of blue light, carvacrol, and existing antibiotics, if proven in vivo, is expected to enhance drug efficacy, curtail disease duration, and improve the quality of life in patients with chronic wounds, otitis media, sinusitis, periodontitis, or chronic infections associated with biofilm-contaminated areas which are accessible by blue light.

Many infectious diseases are polymicrobial such as diabetic foot ulcers and other chronic wounds where skin flora encounter environmental microorganisms. The polymicrobial biofilms we created in wound-like medium turned out to be *P. aeruginosa*-dominated and *E. coli*-deficient. *P. aeruginosa* dominance was also reported elsewhere using a drip-flow polymicrobial biofilm model [17]. *P. aeruginosa* excretes several products, including LasA protease, 4-hydroxy-2-heptylquinoline-N-oxide (HQNO), the *pel* and *psl* products, and pyocyanin to compete with neighboring residents, particularly Gram-positive bacteria [20]. The reason why *E. coli* IQ0245 was exiled from the community remained unclear. Nevertheless, omitting *E. coli* did not change the overall bacterial composition of the biofilm. The resultant four-species biofilm is an excellent model for tolerance study because the biofilm is composed of Gram-positive and Gram-negative, BL-sensitive and refractory, and different antibiotic resistant bacteria. The bactericidal ciprofloxacin mainly targets fast-growing bacteria where inhibition of the Type II/IV topoisomerases rapidly induces massive DNA fragmentations [33]. Conceivably, the ability of ciprofloxacin to enter the cells and interact with the Type II/IV topoisomerases until a poisonous concentration is reached is vital for its bactericidal activity. Biofilm bacteria were known to develop adaptive tolerance to ciprofloxacin and other quinolones by upregulation of efflux pumps to prevent the intracellular accumulation of drugs [22,34]. In line with this, biofilm-associated bacteria were clearly tolerant to ciprofloxacin at supra-MIC (≥ 10 MIC) as compared with the planktonic mixture. On the contrary, tetracycline merely blocks ribosomal protein synthesis, which inhibits, rather than killing, the bacteria. The different bactericidal mechanisms between ciprofloxacin and tetracycline may be the underlying mechanism for distinct responses to BL-Ca dual treatment, although further studies are required to corroborate it. The polymicrobial biofilm was both resistant and tolerant to BL-Ca treatment. The resistant part came from *K. pneumoniae* because *K. pneumoniae* was naturally indifferent to blue light (Figure 3e). Tolerance to the dual treatment was corroborated by a higher survival rate of *P. aeruginosa* in polymicrobial biofilm (2.7 Log reduction) than in planktonic *P. aeruginosa* (>5 Log reduction) (Figure 2a,d). Lu et al. also demonstrated that *P. aeruginosa*, *S. aureus*, and *A. baumannii* mono-species biofilms could tolerate higher doses of BL-Ca than their planktonic counterparts [24].

**Figure 4:**
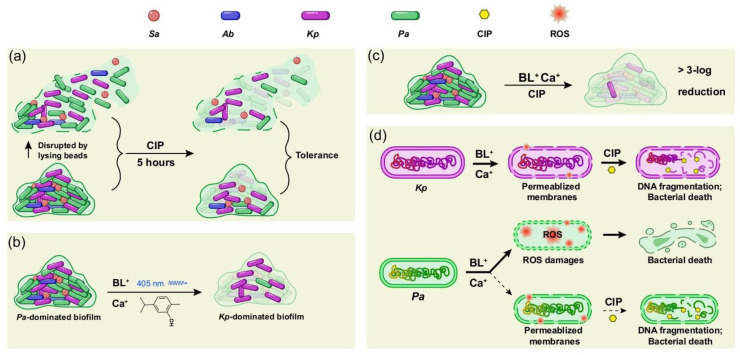
Schematic diagrams of anti-biofilm modalities. (**a**) Intact biofilm or disrupted biofilm showed similar tolerance to ciprofloxacin (CIP). (**b**) *P. aeruginosa*-dominated biofilm became *K. pneumoniae*-dominated after BL-Ca dual treatment. (**c**) The cocktail therapy of 80 J/cm^2^ BL, 125 µg/mL Ca, and 10 µg/mL CIP showed strong anti-biofilm activity against the four-species biofilm. (**d**) BL-Ca treatment compromises the bacterial outer/cytoplasmic membranes of *K. pneumoniae* and allows entrance of CIP into the cells. CIP induces DNA fragmentation and cell death. Unlike *K. pneumoniae*, *P. aeruginosa* is mainly killed by ROS overwhelmingly induced by BL-Ca dual treatment. Bacteria survived the treatment could be further killed by CIP that enters the cells sufficiently following BL-Ca treatment. *Sa*: *S. aureus*; *Ec*: *E. coli*; *Kp*: *K. pneumoniae*; *Ab*: *A. baumannii*; *Pa*: *P. aeruginosa*.

We compare and summarize the anti-biofilm modalities in Figure 4. The biofilm-associated bacteria show similar tolerance to ciprofloxacin before and after mechanical disruption, indicating that the matrix barrier is not a major contributor to the tolerance (Figure 4a). BL-Ca treatment or ciprofloxacin alone is ineffective in killing the biofilm bacteria but can substantially reshape the biofilm composition (i.e., from *P. aeruginosa*-dominated to *K. pneumoniae*-dominated) (Figure 2b and Figure 4a,b). How such a transition will affect the biofilm matrix, structure, and overall infectivity is still not clear. Eradication of 99.9% (3 Log reduction in CFU/mL) of biofilm-embedded bacteria has been suggested as an endpoint parameter to guide treatments of biofilm-associated infections. In this regard, the anti-biofilm activity of ciprofloxacin or BL-Ca treatment alone was far from satisfactory but a combination of the two was successful. BL-Ca treatment and ciprofloxacin synergistically kill the polymicrobial biofilm (Figure 4c). Intriguingly, *P. aeruginosa* and *K. pneumoniae* appear to respond differently to the cocktail treatment (Figure 4d). The *P. aeruginosa* membranes were far more porous than the *K. pneumoniae* membranes after BL-Ca treatment, indicating more robust ROS production in *P. aeruginosa* cells. The fundamental difference between *P. aeruginosa* and *K. pneumoniae* lies in the high susceptibility of *P. aeruginosa* to BL-based treatment [24,27] in contrast to the natural resistance of *K. pneumoniae* to blue light [35,36]. *P. aeruginosa* was found to produced BL-sensitive porphyrins [37,38], while similar reports for *K. pneumoniae* is still unavailable. We propose that the BL-Ca treatment induces ROS production in all four species tested, including the less-sensitive *K. pneumoniae* cells. Albeit inadequate to kill the *K. pneumoniae*, the ROS compromises the bacterial membranes, which encourages ciprofloxacin entry into the cells so that it can interact with intracellular targets sufficiently. The membrane injury may also abolish the efflux pump preventing it from actively pumping ciprofloxacin out of the cells. Ciprofloxacin accumulation induces subsequent DNA fragmentation and cell death (Figure 4d, purple). As for the BL-sensitive *P. aeruginosa*, BL-Ca alone can induce an overwhelming ROS production and kill most of the cells. The remaining fraction of *P. aeruginosa* is not killed by the sub-optimal ROS production probably due to the attenuated penetration of blue light through the biofilm. However, ciprofloxacin is still able to accumulate in these *P. aeruginosa* cells via the impaired membranes and causes bacterial death.

The major limitation of this study was that we only used planktonic cells at the stationary phase to assay the membrane integrities. There could be a substantial difference in membrane structures between the planktonic cells and the biofilm-associated cells [39]. Future experiments that use biofilm subpopulations (e.g., persister cells achieved by cell sorting) to confirm the altered membrane permeability in response to BL, Ca, or BL-Ca treatments will add confidence to the proposed mechanisms.

In summary, this study demonstrates that BL-Ca dual treatment could reverse the polymicrobial biofilm-associated tolerance to existing antibiotics by permeabilizing the bacterial membranes. Anti-biofilm activity is more potent when BL-Ca is combined with the bactericidal ciprofloxacin than the bacteriostatic tetracycline. BL-susceptible bacteria like *P. aeruginosa* are more sensitive to BL-Ca potentiation, probably due to higher ROS production. Future studies on optimizing ROS production in BL-resistant bugs like *K. pneumoniae* or disturbing bacterial antioxidant response may help to enhance antibiotic potency against the recalcitrant biofilms. The cocktail described in the study may serve as a starting point to fabricate antimicrobial wound dressing in the future using light-transmissible materials, which is particularly significant considering that systemic administration of ciprofloxacin proved ineffective against chronic wound biofilms [40]. The transparent wound dressing incorporating carvacrol and ciprofloxacin can be repeatedly and topically applied to the wound to sensitize the recalcitrant biofilm to ciprofloxacin killing while creating a balance humidity and promoting wound healing.

## Figures and Tables

**Table 1:** Species-specific primer sets.

Name	Sequence 5′ to 3′	Tm (°C)	Target Gene	Size (bp)	Source
Sa-F	ATTTGGTCCCAGTGGTGTGGGTAT	60.4	hypothetical protein	143	Sun et al. [18]
Sa-R	GCTGTGACAATTGCCGTTTGTCGT	60.7			
Ec-F	CGCGGTGATACATATCCAGCCA	59.1	*uidA*	179	This study
Ec-R	CGATTTGGAAACGGCAGAGAAGGT	59.6			
Kp-F	GGCTTATACCGATAGAGAACTCGAACTG	58.8	*ureR_1*	162	This study
Kp-R	TCGATAAAGCCATGAGAATGCAACGG	59.4			
Ab-F	GGTTCTTCGCTCCTTTGTCCG	58.3	hypothetical protein	164	Sahl et al. [31]
Ab-R	CTCGGTAATGCTGTTTAACTTCAGGAATATC	57.3			
Pa-F	TAAGGACAGCCAGGACTACGAGAA	59.0	*lasR*	159	Sun et al. [18]
Pa-R	TGGTAGATGGACGGTTCCCAGAAA	59.8			
Universal-F	CACAAGCGGTGGAGCATGT	58.8	16S rRNA gene	145	This study
Universal-R	ACGACACGAGCTGACGACA	58.8			

**Table 2:** MICs of the four common pathogens.

Planktonic Cells	Tetracycline	Ciprofloxacin	Carvacrol
*S. aureus* IQ0064	0.25; S *	1; S	200
*K. pneumoniae* IQ0035	2–4; S	0.13–0.25; S	250
*A. baumannii* 17978	1–2; S	0.13–0.25; S	100
*P. aeruginosa* PAO1	2–4; S	0.06; S	500

* S: Susceptible. Susceptibilities were determined according to the breakpoints by Clinical and Laboratory Standards Institute. MIC unit: µg/mL.

## References

[1] Stewart P.S., Costerton J.W. (2001). Antibiotic resistance of bacteria in biofilms. Lancet.

[2] Grant S.S., Hung D.T. (2013). Persistent bacterial infections, antibiotic tolerance, and the oxidative stress response. Virulence.

[3] Hall C.W., Mah T.-F. (2017). Molecular mechanisms of biofilm-based antibiotic resistance and tolerance in pathogenic bacteria. FEMS Microbiol. Rev..

[4] Shigeta M., Tanaka G., Komatsuzawa H., Sugai M., Suginaka H., Usui T. (1997). Permeation of antimicrobial agents through Pseudomonas aeruginosa biofilms: A simple method. Chemotherapy.

[5] Lewis K. (2008). Multidrug tolerance of biofilms and persister cells. Bact. Biofilms.

[6] Jensen P.Ø., Briales A., Brochmann R.P., Wang H., Kragh K.N., Kolpen M., Hempel C., Bjarnsholt T., Høiby N., Ciofu O. (2014). Formation of hydroxyl radicals contributes to the bactericidal activity of ciprofloxacin against Pseudomonas aeruginosa biofilms. Pathog. Dis..

[7] Reza A., Sutton J.M., Rahman K.M. (2019). Effectiveness of efflux pump inhibitors as biofilm disruptors and resistance breakers in gram-negative (ESKAPEE) bacteria. Antibiotics.

[8] Lewis K. (2001). Riddle of biofilm resistance. Antimicrob. Agents Chemother..

[9] Harriott M.M., Noverr M.C. (2011). Importance of Candida–bacterial polymicrobial biofilms in disease. Trends Microbiol..

[10] Elias S., Banin E. (2012). Multi-species biofilms: Living with friendly neighbors. FEMS Microbiol. Rev..

[11] Wolcott R., Costerton J., Raoult D., Cutler S. (2013). The polymicrobial nature of biofilm infection. Clin. Microbiol. Infect..

[12] James G.A., Swogger E., Wolcott R., Pulcini E.d., Secor P., Sestrich J., Costerton J.W., Stewart P.S. (2008). Biofilms in chronic wounds. Wound Repair Regen..

[13] Dalton T., Dowd S.E., Wolcott R.D., Sun Y., Watters C., Griswold J.A., Rumbaugh K.P. (2011). An in vivo polymicrobial biofilm wound infection model to study interspecies interactions. PLoS ONE.

[14] Hoffman L.R., Déziel E., d’Argenio D.A., Lépine F., Emerson J., McNamara S., Gibson R.L., Ramsey B.W., Miller S.I. (2006). Selection for Staphylococcus aureus small-colony variants due to growth in the presence of Pseudomonas aeruginosa. Proc. Natl. Acad. Sci. USA.

[15] Lebeaux D., Chauhan A., Rendueles O., Beloin C. (2013). From in vitro to in vivo models of bacterial biofilm-related infections. Pathogens.

[16] Ferrer-Espada R., Liu X., Goh X.S., Dai T. (2019). Antimicrobial blue light inactivation of polymicrobial biofilms. Front. Microbiol..

[17] Woods J., Boegli L., Kirker K.R., Agostinho A.M., Durch A.M., Delancey Pulcini E., Stewart P.S., James G.A. (2012). Development and application of a polymicrobial, in vitro, wound biofilm model. J. Appl. Microbiol..

[18] Sun Y., Dowd S.E., Smith E., Rhoads D.D., Wolcott R.D. (2008). In vitro multispecies Lubbock chronic wound biofilm model. Wound Repair Regen..

[19] Sun Y., Smith E., Wolcott R., Dowd S. (2009). Propagation of anaerobic bacteria within an aerobic multi-species chronic wound biofilm model. J. Wound Care.

[20] DeLeon S., Clinton A., Fowler H., Everett J., Horswill A.R., Rumbaugh K.P. (2014). Synergistic interactions of *Pseudomonas aeruginosa* and *Staphylococcus aureus* in an in vitro wound model. Infect. Immun..

[21] Marques C.N., Davies D.G., Sauer K. (2015). Control of biofilms with the fatty acid signaling molecule cis-2-decenoic acid. Pharmaceuticals.

[22] Bjarnsholt T., Ciofu O., Molin S., Givskov M., Høiby N. (2013). Applying insights from biofilm biology to drug development—Can a new approach be developed?. Nat. Rev. Drug Discov..

[23] Sultana S.T., Call D.R., Beyenal H. (2016). Eradication of Pseudomonas aeruginosa biofilms and persister cells using an electrochemical scaffold and enhanced antibiotic susceptibility. NPJ Biofilms Microbiomes.

[24] Lu M., Wang S., Wang T., Hu S., Bhayana B., Ishii M., Kong Y., Cai Y., Dai T., Cui W. (2021). Bacteria-specific phototoxic reactions triggered by blue light and phytochemical carvacrol. Sci. Transl. Med..

[25] Zhang Y., Zhu Y., Gupta A., Huang Y., Murray C.K., Vrahas M.S., Sherwood M.E., Baer D.G., Hamblin M.R., Dai T. (2014). Antimicrobial blue light therapy for multidrug-resistant Acinetobacter baumannii infection in a mouse burn model: Implications for prophylaxis and treatment of combat-related wound infections. J. Infect. Dis..

[26] Hamblin M.R., Viveiros J., Yang C., Ahmadi A., Ganz R.A., Tolkoff M.J. (2005). Helicobacter pylori accumulates photoactive porphyrins and is killed by visible light. Antimicrob. Agents Chemother..

[27] Lu M., Li Y., Wu M.X. (2021). Bacteria-specific pro-photosensitizer kills multidrug-resistant *Staphylococcus aureus* and *Pseudomonas aeruginosa*. Commun. Biol..

[28] Halstead F.D., Thwaite J.E., Burt R., Laws T.R., Raguse M., Moeller R., Webber M.A., Oppenheim B.A. (2016). Antibacterial activity of blue light against nosocomial wound pathogens growing planktonically and as mature biofilms. Appl. Environ. Microbiol..

[29] Lu M., Dai T., Murray C.K., Wu M.X. (2018). Bactericidal property of oregano oil against multidrug-resistant clinical isolates. Front. Microbiol..

[30] Courtney C.M., Goodman S.M., Nagy T.A., Levy M., Bhusal P., Madinger N.E., Detweiler C.S., Nagpal P., Chatterjee A. (2017). Potentiating antibiotics in drug-resistant clinical isolates via stimuli-activated superoxide generation. Sci. Adv..

[31] Sahl J.W., Johnson J.K., Harris A.D., Phillippy A.M., Hsiao W.W., Thom K.A., Rasko D.A. (2011). Genomic comparison of multi-drug resistant invasive and colonizing Acinetobacter baumannii isolated from diverse human body sites reveals genomic plasticity. BMC Genom..

[32] Wu H., Moser C., Wang H.-Z., Høiby N., Song Z.-J. (2015). Strategies for combating bacterial biofilm infections. Int. J. Oral Sci..

[33] Silva F., Lourenço O., Queiroz J.A., Domingues F.C. (2011). Bacteriostatic versus bactericidal activity of ciprofloxacin in *Escherichia coli* assessed by flow cytometry using a novel far-red dye. J. Antibiot..

[34] Zhang L., Mah T.-F. (2008). Involvement of a novel efflux system in biofilm-specific resistance to antibiotics. J. Bacteriol..

[35] Dos Anjos C., Sabino C.P., Sellera F.P., Esposito F., Pogliani F.C., Lincopan N. (2020). Hypervirulent and hypermucoviscous strains of Klebsiella pneumoniae challenged by antimicrobial strategies using visible light. Int. J. Antimicrob. Agents.

[36] Hoenes K., Bauer R., Meurle T., Spellerberg B., Hessling M. (2021). Inactivation Effect of Violet and Blue Light on ESKAPE Pathogens and Closely Related Non-pathogenic Bacterial Species—A Promising Tool against Antibiotic-Sensitive and Antibiotic-Resistant Microorganisms. Front. Microbiol..

[37] Amin R.M., Bhayana B., Hamblin M.R., Dai T. (2016). Antimicrobial blue light inactivation of Pseudomonas aeruginosa by photo-excitation of endogenous porphyrins: In vitro and in vivo studies. Lasers Surg. Med..

[38] Wang Y., Wu X., Chen J., Amin R., Lu M., Bhayana B., Zhao J., Murray C.K., Hamblin M.R., Hooper D.C. (2016). Antimicrobial blue light inactivation of gram-negative pathogens in biofilms: In vitro and in vivo studies. J. Infect. Dis..

[39] Dubois-Brissonnet F., Trotier E., Briandet R. (2016). The biofilm lifestyle involves an increase in bacterial membrane saturated fatty acids. Front. Microbiol..

[40] Hill K.E., Malic S., McKee R., Rennison T., Harding K.G., Williams D.W., Thomas D.W. (2010). An in vitro model of chronic wound biofilms to test wound dressings and assess antimicrobial susceptibilities. J. Antimicrob. Chemother..

